# Supplementary material for: Transcriptional profiling of Hutchinson-Gilford progeria patients identifies primary target pathways of progerin
Source: Nucleus. 2026 Jan 5;17(1):2611484. doi: 10.1080/19491034.2025.2611484 (PMC12773485; doi:10.1080/19491034.2025.2611484)
Supplement: SUPPLEMENTAL MATERIAL.docx [file KNCL_A_2611484_SM4987.docx]

# Figure S1

## A


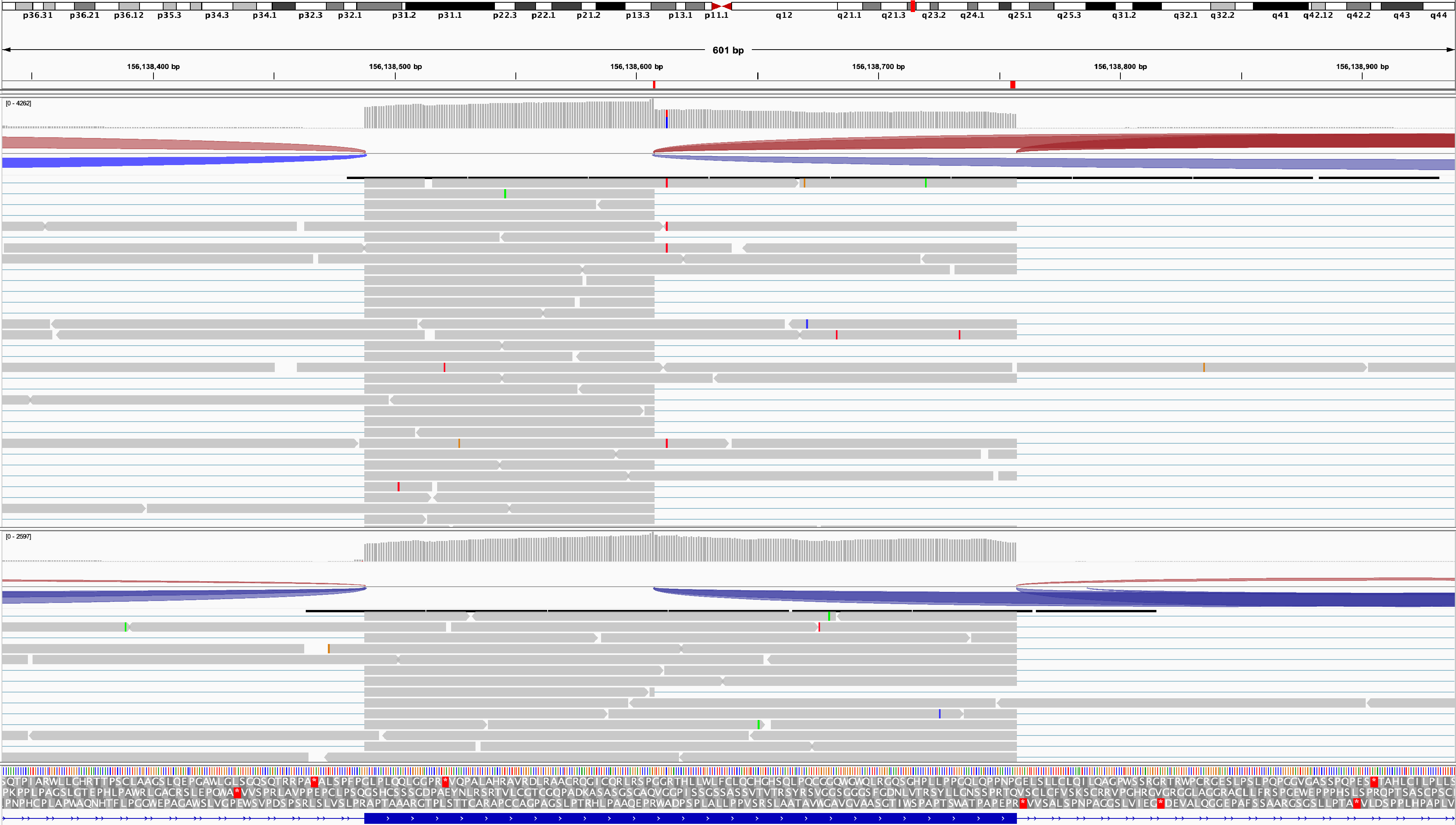


**HGPS8**

**Progerin splice site**

**Lamin A splice site**

**WT4**

***LMNA***

**Sequence**

***Zmpste24 c*leavage site**

## B


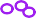

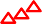

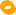

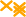

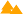

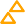

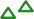

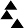

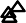


**30**

**20**

**PC2: 10% variance**

**10**

**0**

**−10**

**−20**

**WT1 WT2 WT3 WT4 WT5**

**WT6 WT7 WT8 WT9 WT10**

**HGPS1 HGPS2 HGPS3 HGPS4 HGPS5**

**HGPS6 HGPS7 HGPS8 HGPS9 HGPS10**

**HGPS11 HGPS12 HGPS14 HGPS15**

**HGPS16 HGPS18**

**HGPS13 HGPS17 HGPS19**


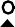


**HGPS20**

**−30**

**−20 0 20 40**

**PC1: 29% variance**

**C PRF**


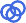

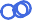

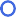

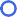

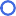

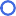

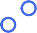

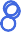

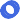

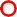

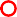

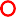

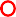

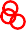

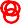

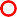

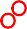

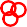

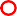

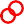

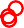

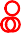

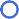

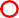


**WT**

**HGPS**

**20**

**CORIELL**

**40**


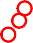

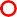

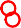

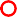

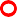

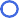

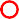


**WT**

**HGPS**

**20**

**PC2: 14% variance**

**PC2: 19% variance**

**0**

**−20 0**

**−40**

**−40 −20 0 20**

**PC1: 35% variance**

**−20**

**−25 0 25 50**

**PC1: 40% variance**

### Figure S1 Alt Text:

**Figure S1A:** Integrative Genomics Viewer representation of the splice sites for progerin and lamin A.

**Figure S1B:** Scatter plot of the two principal components from gene expression analysis of all control and HGPS samples, including non-classical HGPS. Most HGPS samples cluster separately from controls, while non-classical HGPS patients cluster with wild type controls.

**Figure S1C:** Scatter plot of the two principal components of gene expression analysis of control and classic HGPS samples separated by source. Samples from the Progeria Research Foundation are displayed on the left, while samples from the Coriell repository are shown on the right.

**Figure S1. Characterization of primary human dermal fibroblasts. (A)** Integrative Genomics Viewer (IGV) screen capture showing the two splicing junctions at chr1:156,138,607 (progerin) and chr: 156,138,757 (Lamin A). Sequence reads at these sites were used to quantitate progerin and wild type lamin A transcripts in a control and HGPS patient. **(B)** Principal component analysis (PCA) of the primary fibroblast datasets used in this study. Samples primarily segregate by progeria/control phenotype with four samples classified by the NIA Aging Cell Repository (Coriell) as progeria patients showing relatively similar gene expression patterns as the WT samples (black symbols). Follow-up analysis demonstrated absence of the classic c.1824C>T mutation in these samples and identified them as atypical progerias. These samples were excluded from analysis. Top 500 genes with the highest variability across samples were used for PCA plot. All samples were analyzed in triplicate. **(C)** PCA of the primary fibroblast datasets based on the source (PRF Cell and Tissue Bank, Coriell NIA Aging Cell Repository). Samples primarily

segregate by progeria/control phenotype. Blue dots represent wild type control samples and red dots represent progeria patients. All samples were analyzed in triplicate.

# Figure S2

##
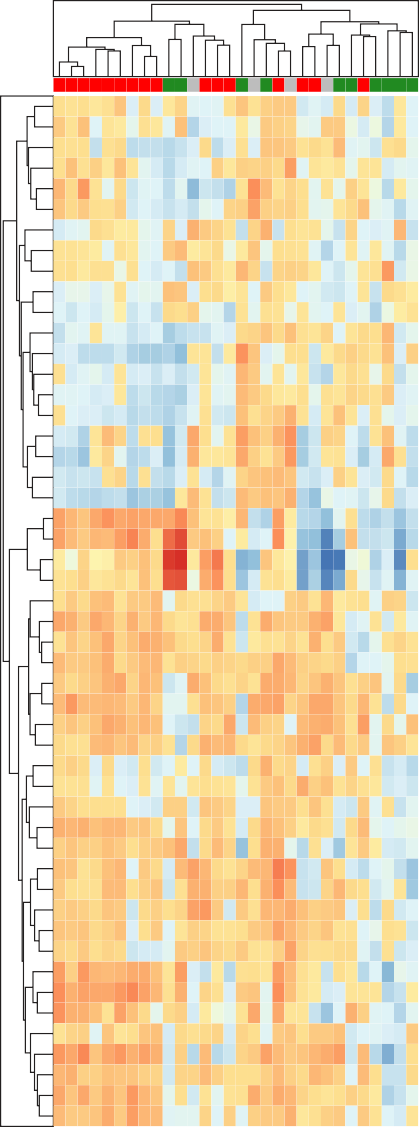
B

**
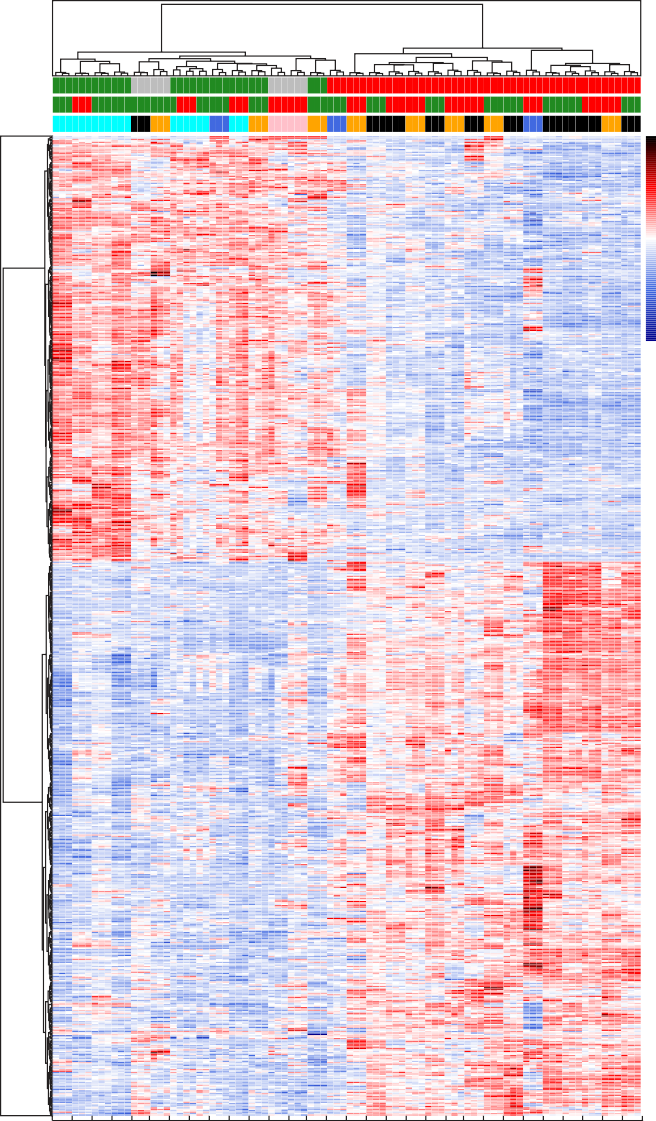
Treatment Sex**

**Age**

**Treatment**

**Treatment ANDROGEN_RESPONSE TGF_BETA_SIGNALING COAGULATION**

**
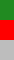

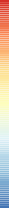
3 Treatment**

**WT**

**2 HGPS**

**Non−classical**

**
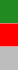
4 WT**

**2 HGPS**

**Non−classical HGPS**

**
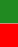
0 Sex**

**−2 F**

**M**

**REACTIVE_OXYGEN_SPECIES_PATHWAY 1**

**PROTEIN_SECRETION**

**ADIPOGENESIS 0**

**APICAL_SURFACE −1**

**DNA_REPAIR**

**CHOLESTEROL_HOMEOSTASIS −2**

**SPERMATOGENESIS −3**

**PANCREAS_BETA_CELLS**

**HGPS**

**
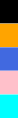
−4 Age**

**1<age<=5 5<age<=10 10<age<=15 15<age<=20 20<age**

**HEME_METABOLISM BILE_ACID_METABOLISM FATTY_ACID_METABOLISM PEROXISOME XENOBIOTIC_METABOLISM INTERFERON_GAMMA_RESPONSE INTERFERON_ALPHA_RESPONSE COMPLEMENT IL6_JAK_STAT3_SIGNALING MYC_TARGETS_V2 MYC_TARGETS_V1**

**E2F_TARGETS G2M_CHECKPOINT WNT_BETA_CATENIN_SIGNALING NOTCH_SIGNALING HEDGEHOG_SIGNALING UV_RESPONSE_UP ALLOGRAFT_REJECTION APICAL_JUNCTION**

**MYOGENESIS KRAS_SIGNALING_DN UV_RESPONSE_DN P53_PATHWAY ESTROGEN_RESPONSE_LATE PI3K_AKT_MTOR_SIGNALING MITOTIC_SPINDLE TNFA_SIGNALING_VIA_NFKB INFLAMMATORY_RESPONSE KRAS_SIGNALING_UP APOPTOSIS IL2_STAT5_SIGNALING UNFOLDED_PROTEIN_RESPONSE MTORC1_SIGNALING OXIDATIVE_PHOSPHORYLATION ANGIOGENESIS**

**EPITHELIAL_MESENCHYMAL_TRANSITION ESTROGEN_RESPONSE_EARLY GLYCOLYSIS**

**HYPOXIA**

**WT9 WT2 WT6 WT10 HGPS14 WT7 WT1 HGPS20 HGPS9 HGPS16 HGPS17 HGPS18 WT8 HGPS13 WT5 HGPS7 HGPS10 HGPS6 HGPS19 WT4 WT3 HGPS3 HGPS1 HGPS4 HGPS8 HGPS2 HGPS5 HGPS15 HGPS11 HGPS12**

**HGPS4 HGPS5 HGPS1**

**HGPS3 HGPS2 HGPS18 HGPS10**

**HGPS9 HGPS16**

**HGPS15**

**HGPS12 HGPS8 HGPS11 HGPS6 HGPS7**

**HGPS14**

**WT7 HGPS20 HGPS13**

**WT9 WT10 WT8 WT3**

**WT4 HGPS17 HGPS19**

**WT5**

**WT1**

**WT2**

**WT6**

### Figure S2 Alt Text:

**Figure S2A:** Hierarchical clustered heatmap of gene expression in wild type and all HGPS samples revealing clustering of non-classic HGPS patients with wild type control.

**Figure S2B:** Heatmap displaying GSEA Hallmark pathways showing positive or negative enrichment in all HGPS patients, including non-classic HGPS. Each row represents one pathway, and columns represent individual samples.

**Figure S2. Pathway analysis in atypical progeria patients. (A)** Heatmap of RNA-seq transcriptome analysis for DEGs consistently up- or down-regulated in 13 or more patients (80%) for all 20 patients, including atypical progeria samples (gray). Heatmap of z-scaled, variance-stabilized gene expression values were generated using the Euclidean distance and Ward.D2 clustering method. All samples were analyzed in triplicate. **(B)** Heatmap of 50 GSEA hallmark pathways positively or negatively enriched in all 20 HGPS patients. Atypical progeria samples are shown in gray. Heatmap of mean-centered NES values was generated using Euclidean distance and complete linkage clustering method.

# Figure S3

##
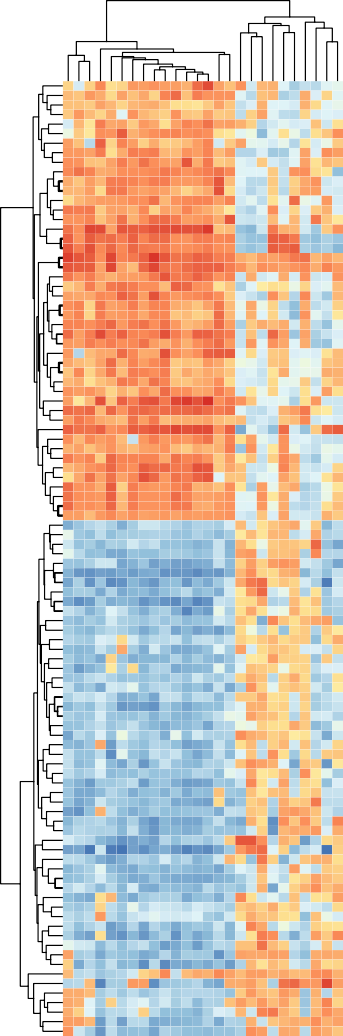

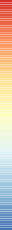
A _2_

**1**

**B_CELL_HOMEOSTASIS NEGATIVE_REGULATION_OF_TOLL_LIKE_RECEPTOR_4_SIGNALING_PATHWAY REGULATION_OF_ERAD_PATHWAY**

**0**

**MACROMOLECULE_DEACYLATION POSITIVE_REGULATION_OF_IRE1_MEDIATED_UNFOLDED_PROTEIN_RESPONSE ER_ASSOCIATED_MISFOLDED_PROTEIN_CATABOLIC_PROCESS POSITIVE_REGULATION_OF_MAST_CELL_ACTIVATION PERICENTRIC_HETEROCHROMATIN_FORMATION LINOLEIC_ACID_METABOLIC_PROCESS PHOSPHATIDYLSERINE_EXPOSURE_ON_APOPTOTIC_CELL_SURFACE ORGANELLE_TRANSPORT_ALONG_MICROTUBULE VESICLE_TRANSPORT_ALONG_MICROTUBULE APOPTOTIC_PROCESS_INVOLVED_IN_BLOOD_VESSEL_MORPHOGENESIS RESPONSE_TO_INSECTICIDE**

**-1**

**-2**

**REGULATION_OF_VENTRICULAR_CARDIAC_MUSCLE_CELL_MEMBRANE_REPOLARIZATION BASEMENT_MEMBRANE_ORGANIZATION**

**CELL_ADHESION CELL_CELL_ADHESION**

**REGULATION_OF_CARDIOCYTE_DIFFERENTIATION POSITIVE_REGULATION_OF_CARDIOCYTE_DIFFERENTIATION NEGATIVE_REGULATION_OF_VASCULAR_ASSOCIATED_SMOOTH_MUSCLE_CELL_MIGRATION REGULATION_OF_SYNAPTIC_VESICLE_CLUSTERING**

**MAINTENANCE_OF_PROTEIN_LOCATION MACROMOLECULE_DEPALMITOYLATION PROTEIN_DEPALMITOYLATION**

**REGULATION_OF_ENDOSOME_TO_PLASMA_MEMBRANE_PROTEIN_TRANSPORT CELL_ADHESION_MEDIATED_BY_INTEGRIN NEUROTRANSMITTER_GATED_ION_CHANNEL_CLUSTERING MULTICELLULAR_ORGANISMAL_MOVEMENT**

**ATP_TRANSPORT ADP_TRANSPORT MEIOTIC_CYTOKINESIS PROTEIN_LIPOYLATION GLUCOSE_CATABOLIC_PROCESS**

**ENGULFMENT_OF_APOPTOTIC_CELL MEMBRANE_INVAGINATION NTERMEDIATE_FILAMENT_BUNDLE_ASSEMBLY NEURONAL_ACTION_POTENTIAL_PROPAGATION CARDIAC_FIBROBLAST_CELL_DIFFERENTIATION**

**BRAIN_DERIVED_NEUROTROPHIC_FACTOR_RECEPTOR_SIGNALING_PATHWAY**

1. **KRAS_SIGNALING_DN**

**
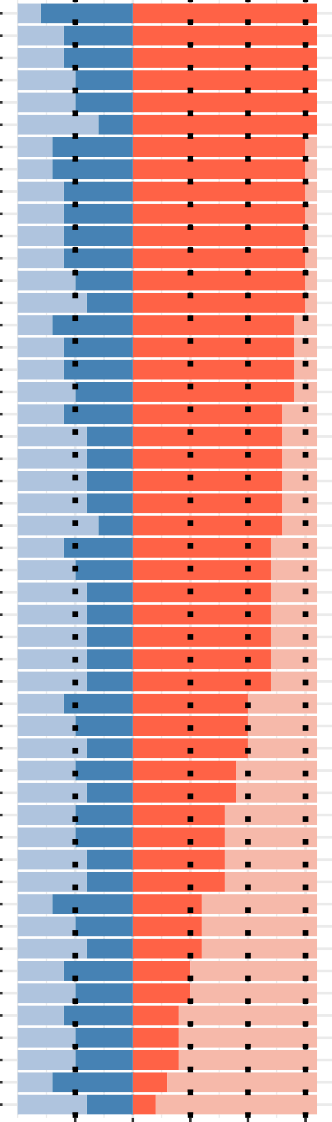
ALLOGRAFT_REJECTION ESTROGEN_RESPONSE_EARLY**

**APICAL_JUNCTION MYOGENESIS PI3K_AKT_MTOR_SIGNALING HEDGEHOG_SIGNALING UV_RESPONSE_UP MTORC1_SIGNALING**

**GLYCOLYSIS NOTCH_SIGNALING WNT_BETA_CATENIN_SIGNALING**

**MITOTIC_SPINDLE HALLMARK_HYPOXIA**

**APOPTOSIS INFLAMMATORY_RESPONSE KRAS_SIGNALING_UP EPITHELIAL_MESENCHYMAL_TRANSITION UNFOLDED_PROTEIN_RESPONSE**

**MYC_TARGETS_V2 G2M_CHECKPOINT**

**NEGATIVE_REGULATION_OF_MITOCHONDRIAL_OUTER_MEMBRANE_PERMEABILIZATION_INVOLVED_IN_APOPTOTIC_SIGNALING_PATHWAY REGULATION_OF_MITOCHONDRIAL_OUTER_MEMBRANE_PERMEABILIZATION_INVOLVED_IN_APOPTOTIC_SIGNALING_PATHWAY NEURAL_PLATE_PATTERN_SPECIFICATION**

**NEURAL_PLATE_REGIONALIZATION NEURAL_PLATE_ANTERIOR_POSTERIOR_REGIONALIZATION MITOCHONDRIAL_ADP_TRANSMEMBRANE_TRANSPORT REGULATION_OF_HORMONE_BIOSYNTHETIC_PROCESS CHITIN_METABOLIC_PROCESS**

**MUCOCILIARY_CLEARANCE CEREBROSPINAL_FLUID_CIRCULATION**

**POSITIVE_REGULATION_OF_ENDOTHELIAL_CELL_APOPTOTIC_PROCESS AMINE_CATABOLIC_PROCESS INTERLEUKIN_6_MEDIATED_SIGNALING_PATHWAY THYROID_HORMONE_MEDIATED_SIGNALING_PATHWAY PROGESTERONE_METABOLIC_PROCESS NEGATIVE_REGULATION_OF_B_CELL_MEDIATED_IMMUNITY UTERINE_SMOOTH_MUSCLE_CONTRACTION REGULATION_OF_ER_TO_GOLGI_VESICLE_MEDIATED_TRANSPORT GOBP_RESPONSE_TO_EPIDERMAL_GROWTH_FACTOR REGULATION_OF_CELL_CELL_ADHESION_MEDIATED_BY_INTEGRIN POSITIVE_REGULATION_OF_LEUKOCYTE_TETHERING_OR_ROLLING REGULATION_OF_RETROGRADE_TRANSPORT_ENDOSOME_TO_GOLGI GASTRIC_MOTILITY NEGATIVE_REGULATION_OF_TYPE_2_IMMUNE_RESPONSE CELLULAR_RESPONSE_TO_GLUCOCORTICOID_STIMULUS CELLULAR_RESPONSE_TO_CORTICOSTEROID_STIMULUS MACROPHAGE_PROLIFERATION**

**NEGATIVE_REGULATION_OF_TRANSCRIPTION_FROM_RNA_POLYMERASE_II_PROMOTER_IN_RESPONSE_TO_STRESS PEPTIDYL_SERINE_AUTOPHOSPHORYLATION**

**NEGATIVE_REGULATION_OF_INTERLEUKIN_6_PRODUCTION REGULATION_OF_TOLERANCE_INDUCTION NAD_BIOSYNTHESIS_VIA_NICOTINAMIDE_RIBOSIDE_SALVAGE_PATHWAY MICROGLIAL_CELL_PROLIFERATION NEGATIVE_REGULATION_OF_IMMUNOGLOBULIN_PRODUCTION REGULATION_OF_LEUKOCYTE_TETHERING_OR_ROLLING POSITIVE_REGULATION_OF_STEROID_HORMONE_SECRETION C21_STEROID_HORMONE_METABOLIC_PROCESS NEGATIVE_REGULATION_OF_MAST_CELL_ACTIVATION NEGATIVE_REGULATION_OF_MAST_CELL_ACTIVATION_INVOLVED_IN_IMMUNE_RESPONSE SEGMENT_SPECIFICATION**

**RESPONSE_TO_INTERLEUKIN_6 PEROXISOME_PROLIFERATOR_ACTIVATED_RECEPTOR_SIGNALING_PATHWAY REGULATION_OF_RETINOIC_ACID_RECEPTOR_SIGNALING_PATHWAY NEGATIVE_REGULATION_OF_RETINOIC_ACID_RECEPTOR_SIGNALING_PATHWAY NEGATIVE_REGULATION_OF_MAST_CELL_DEGRANULATION REGULATION_OF_FAT_CELL_PROLIFERATION NEGATIVE_REGULATION_OF_LIPID_BIOSYNTHETIC_PROCESS POSITIVE_REGULATION_OF_SIGNAL_TRANSDUCTION_BY_P53_CLASS_MEDIATOR TYPE_I_PNEUMOCYTE_DIFFERENTIATION**

**CYCLOOXYGENASE_PATHWAY REGULATION_OF_LIPID_CATABOLIC_PROCESS SEX_DIFFERENTIATION MESODERMAL_CELL_FATE_SPECIFICATION POSITIVE_REGULATION_OF_HEART_GROWTH PROXIMAL_DISTAL_PATTERN_FORMATION**

**NEGATIVE_REGULATION_OF_CANONICAL_WNT_SIGNALING_PATHWAY POSITIVE_REGULATION_OF_FATTY_ACID_OXIDATION POSITIVE_REGULATION_OF_LIPID_CATABOLIC_PROCESS MAMMARY_GLAND_EPITHELIUM_DEVELOPMENT MALE_GENITALIA_DEVELOPMENT**

**WT4 WT3 WT9 WT7 WT2 WT1 WT8 WT6 WT5 WT10 HGPS7 HGPS14 HGPS2 HGPS1 HGPS4 HGPS3 HGPS8 HGPS12 HGPS5 HGPS11 HGPS15 HGPS16 HGPS18 HGPS9 HGPS10 HGPS6**

**ANGIOGENESIS**

**ESTROGEN_RESPONSE_LATE CHOLESTEROL_HOMEOSTASIS IL2_STAT5_SIGNALING**

**P53_PATHWAY MYC_TARGETS_V1 E2F_TARGETS TNFA_SIGNALING_VIA_NFKB OXIDATIVE_PHOSPHORYLATION**

**REACTIVE_OXYGEN_SPECIES_PATHWAY**

**ANDROGEN_RESPONSE**

**DNA_REPAIR ADIPOGENESIS COAGULATION APICAL_SURFACE TGF_BETA_SIGNALING**

**INTERFERON_GAMMA_RESPONSE**

**UV_RESPONSE_DN PROTEIN_SECRETION SPERMATOGENESIS INTERFERON_ALPHA_RESPONSE FATTY_ACID_METABOLISM**

**PEROXISOME HEME_METABOLISM BILE_ACID_METABOLISM**

**COMPLEMENT XENOBIOTIC_METABOLISM PANCREAS_BETA_CELLS IL6_JAK_STAT3_SIGNALING**

**5 0 5 10 15**

**Count**


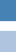
 **WT (NES<0)
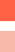
**

**WT (NES>0)**

**HGPS (NES>0)**

HGPS (NES<0)

### Figure S3 Alt Text:

**Figure S3A:** Clustered heatmap of differentially enriched GOBP pathways from GSEA analysis. Normalized Enrichment Scores (NES) for each pathway were compared between HGPS and control samples, with positively enriched pathways shown in red and negatively enriched pathways in blue.

**Figure S3B:** Horizontal bar graph for GSEA Hallmark pathways illustrating the number of HGPS and wild type individuals with positive or negative pathway enrichment.

**Figure S3. Gene ontology (GO) biological processes affected in HGPS patients. (A)** Heatmap of top 100 differentially enriched GOBP GSEA pathways. For each pathway, Normalized Enrichment Score (NES) values were compared between HGPS samples and control samples using a two-group t-test. Pathways were ranked by p-value, and NES values. Top 100 pathways (p<0.005, FDR<0.16) were visualized as a clustered heatmap using Euclidean distance and the complete linkage clustering method.) **(B)** Number of HGPS or WT individuals with NES >0 or NES < 0 for 50 GSEA hallmark pathways. GSEA NES values were obtained using average gene changes in HGPS compared to WT.

# Figure S4

## A B


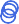

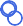

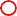

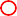

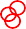

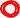

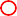

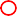

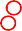

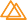

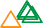

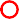

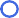

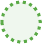

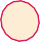


**GFP - progerin OFF**

**GFP - progerin ON**

***+ Dox***

***6 days***

**WT**

**HGPS**

**GFP-progerin OFF**

**50 GFP-progerin ON**

**25**

**PC2: 21% variance**

**0**

**−25**

**−40 −20 0 20**

**PC1: 24% variance**

## D


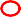

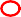

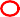

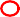

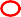

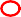

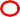

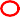

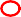

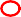

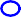

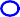

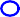

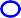

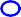

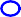

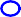

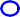

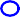


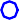
 **WT**


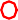
 **HGPS**

**3**

**GFP - progerin ON**

**PC 2: 16% variance**

**0.0**

**0.2**

**Inducible cell line (NES)**

**0**

**1**

**2**

**-0.4**

**-0.4**

**-0.2**

**-2**

**-1**

**-0.3**

**-0.2**

**0.1**

**0.0 0.1**

**0.2**

**-2 -1 0 1 2 3**

**PC 1: 25% variance HGPS (NES)**

### Figure S4 Alt Text:

**Figure S4A:** Schematic representation of GFP-progerin induction in stable human dermal fibroblast cell line.

**Figure S4B:** Scatter plot of two principal components from gene expression analysis comparing primary fibroblasts and GFP-progerin inducible cell lines. The inducible cell lines cluster separately from primary fibroblasts, indicating distinct transcriptional profiles.

**Figure S4C:** Scatter plot of two principal components based on mean normalized enrichment scores (NES) of Hallmark pathways in primary fibroblasts and GFP-progerin inducible cell lines. The GFP-progerin cell line clusters closer to HGPS samples than to wild-type controls, reflecting pathway changes in the progerin cell line that resemble those in HGPS patients.

**Figure S4D:** Scatter plot of NES values for Hallmark pathways in primary fibroblasts and GFP-progerin inducible cell lines, indicating pathways significantly affected in both GFP-progerin expressing cell line and in HGPS patients.

**Figure S4. Comparison between primary patient fibroblasts and GFP-progerin inducible cell line. (A)** Schematic representation of stable human dermal fibroblasts cell line containing doxycycline-inducible GFP-tagged progerin. For analysis, GFP-progerin was expressed for 6 days. Created in BioRender. Vidak, S. (2025) [https://BioRender.com/d40pv1i](https://biorender.com/d40pv1i). **(B)** PCA of the primary

fibroblast and the GFP-progerin inducible cell line datasets used in this study. GFP-progerin cells show a largely different transcriptome compared to HGPS patients or control samples. The top 500 genes with the highest variability across samples were used for PCA plot. Orange triangles represent uninduced GFP-progerin cell line, green triangles represent GFP-progerin cell line where progerin was expressed for 6 days, blue dots represent wild type control samples, and red dots

represent HGPS patients. All samples were analyzed in triplicate. **(C)** PCA plot using mean-centered NES values of 50 hallmark pathways in primary fibroblasts vs. GFP-progerin inducible cell line. Pathway scores of GFP-progerin cell line are more similar to HGPS than the WT samples. Blue circles represent healthy wild type control samples, red circles represent HGPS patients and green triangles represent GFP-progerin cell line where progerin was expressed for 6 days. **(D)** NES scatter plot for 50 GSEA pathways. HGPS NES were obtained using average HGPS gene changes compared to all WT samples for 50 hallmark pathways. Magenta squares indicate 10 pathways significantly affected (padj < 0.05) with consistent positive or negative trends in both GFP-progerin expressing cell line and in HGPS patients. Black circles indicate pathways affected only in HGPS or GFP-progerin expressing cells.

## SUPPLEMENTAL TABLES

### Table S1. HALLMARK PATHWAYS IN HGPS PATIENTS.

**Table S2. GO_BP PATHWAYS IN HGPS PATIENTS.** NES values for each patient are shown in each column.

### Table S3. HALLMARK PATHWAYS IN GFP-PROGERIN CELLS.

**Table S4. GO_BP PATHWAYS IN GFP-PROGERIN CELLS AND HGPS PATIENTS.** NES

values for each patient and GFP-progerin cell line are shown in each column.
